# Supplementary material for: Genome-scale pan-cancer interrogation of lncRNA dependencies using CasRx
Source: Nat Methods. 2024 Feb 26;21(4):584–96. doi: 10.1038/s41592-024-02190-0 (PMC11009108; doi:10.1038/s41592-024-02190-0)
Supplement: Supplementary file 1 — Supplementary Figs. 1–7. [file 41592_2024_2190_MOESM1_ESM.pdf]

nature methods

Article

https://doi.org/10.1038/s41592-024-02190-0

Genome-scale pan-cancer interrogation of lncRNA dependencies using CasRx

In the format provided by the
authors and unedited

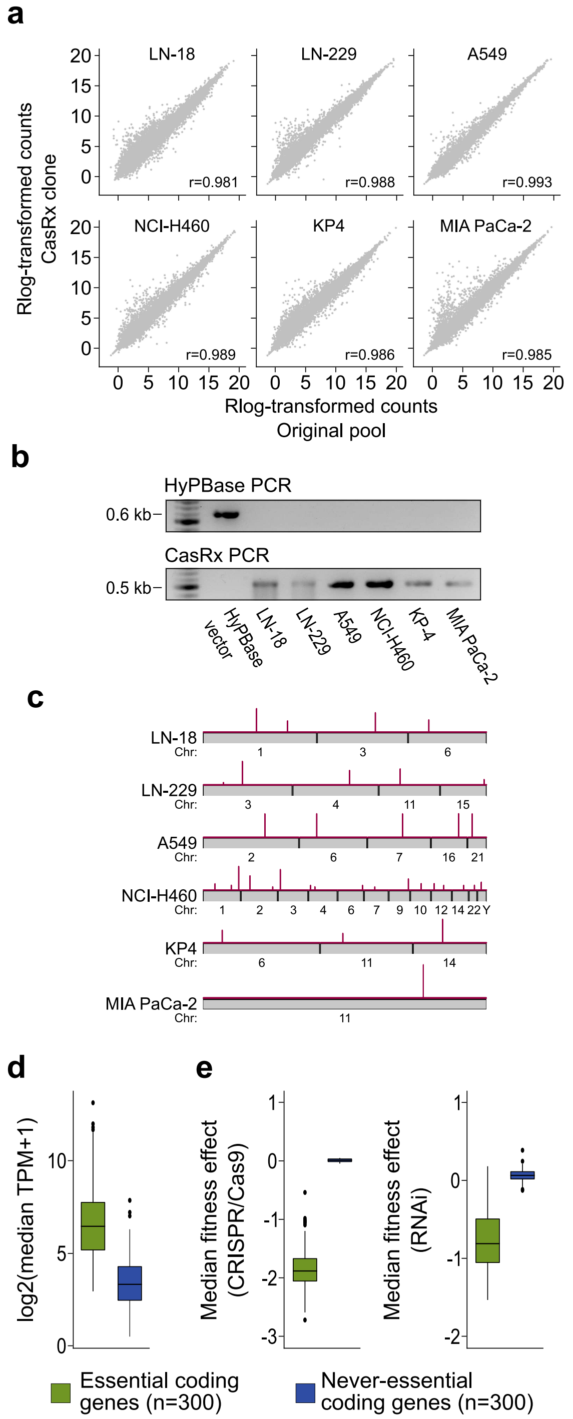

Supplementary Figure 1: Optimization of a genome-integrated CasRx system.

a, Scatter plots showing Rlog-transformed counts comparing RNA-seq data between the original cell lines and their corresponding CasRx clones. **b,** Agarose gel electrophoresis displaying the PCR amplification of the HypBase-transposase or the CasRx-transposon locus using either the HypBase-transposase vector or DNA extracted from the CasRx clones as template. The PCR was performed one time. **c,** Genomic localization of the CasRx-transposon insertions identified using quantitative transposon insertion site sequencing (QiSeq) for all CasRx clones. **d,** Box plot showing the median expression across CCLE cell lines for the never-essential coding genes (blue) or the essential coding genes (green) included in the control library. n=genes. **e,** Box plot showing the median fitness effect across CCLE cell lines for the never-essential coding genes (blue) or the essential coding genes (green) included in the control library. Data have been obtained from both CRISPR/Cas9 (right) or siRNA (left) screens. n=genes. For all the Box plot figures, the data is presented as follows: centre: median; box bounds: 25% and 75% percentile; whisker: 1.5x of inter-quartile range (IQR). Outliers are marked as independent dots.

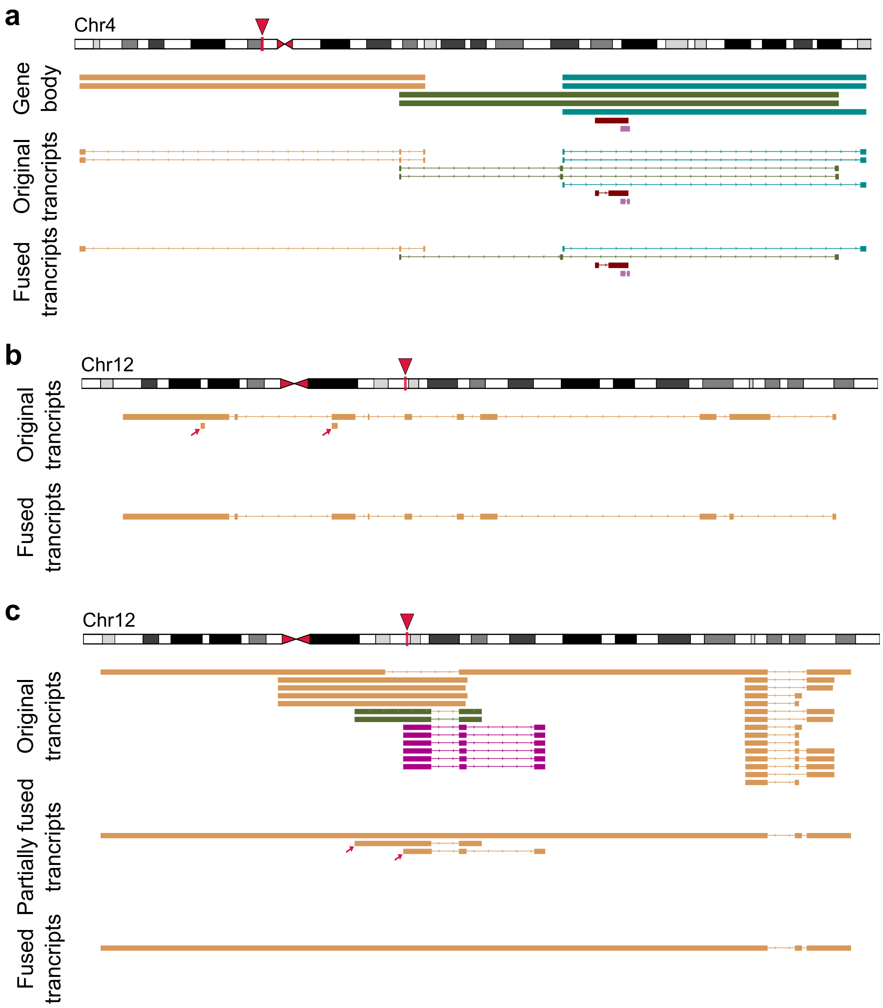

Supplementary Figure 2: Examples of each sequential step used to classify lncRNA transcripts into genes.

a, (i) Only isoforms transcribed from the same strand were processed together. (ii) Isoforms were clustered into genes if they shared genomic localization (transcript body overlap of ≥60%) and they shared at least one exon (exon overlap of ≥60%). **b,** (iii) Mono-exonic transcripts with ≥30% overlap with a gene from **(a)** were included in the same gene. **c,** (iv) Multi-exonic transcripts with ≥ 90% overlap of their total sequence with a gene from **(b)** were included in the same gene.

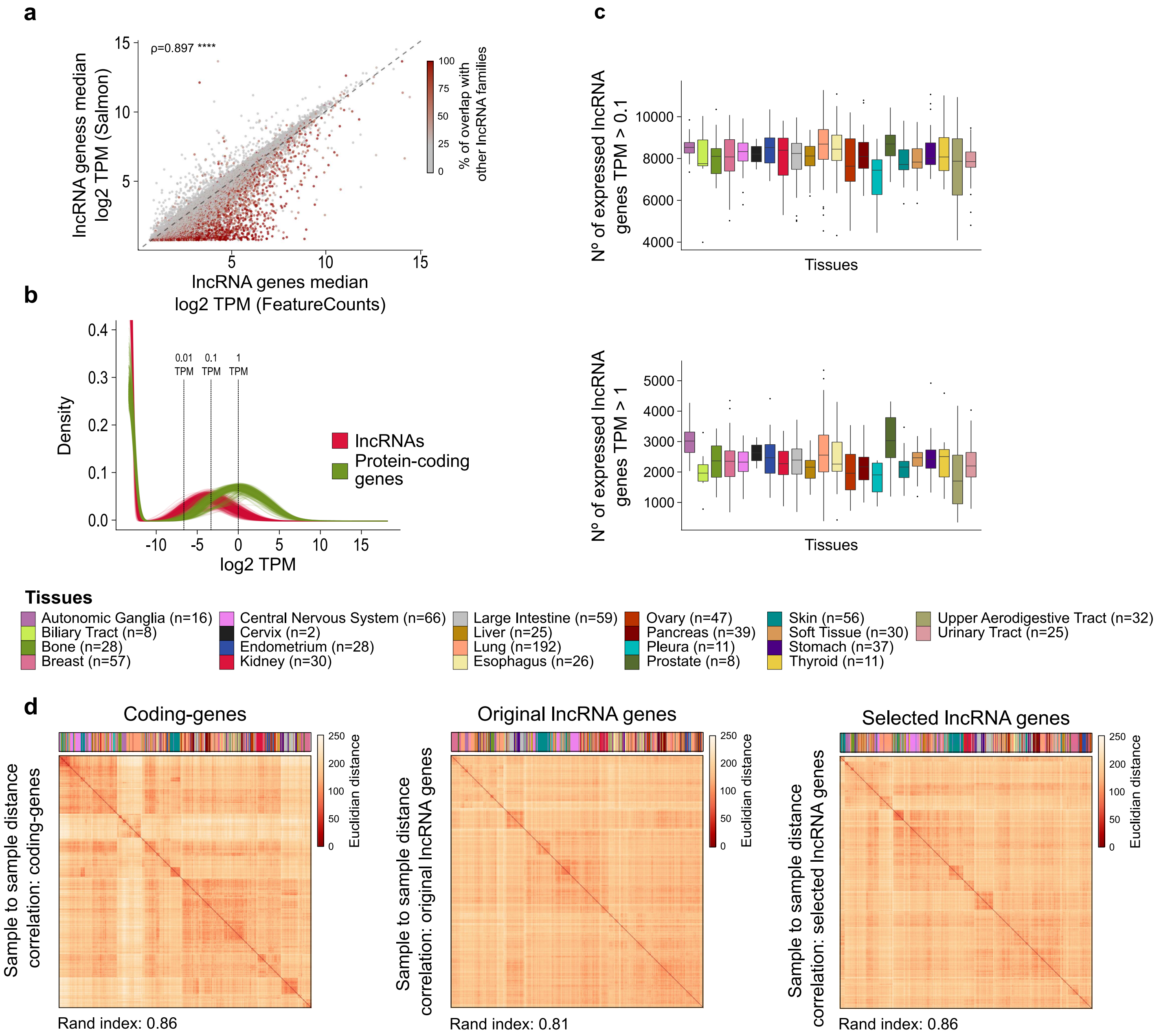

Supplementary Figure 3: Expression calculation and hierarchical clustering of the lncRNA genes included in the Albarossa library.

a, Scatter plots showing the median log2TPM values across the CCLE cell lines of the lncRNA genes included in Albarossa library calculated with two methods: A first "sensitive/permissive" approach (STAR in combination with featureCounts), which served as the basis for selection of lncRNA genes for CRISPR library design. The key goal at this stage was to not penalize the expression quantification of overlapping lncRNAs. In addition, a second method for more "stringent" quantification of lncRNA expression, which served as a basis for a calculation of the number of expressed lncRNA per cell line (Salmon). The percentage of overlap of each particular lncRNA gene with other genes is indicated with a gradient of color (grey to red). Correlation value and significance was calculated using the Spearman regression method. **b,** Density plot showing the log2TPM for the CCLE cell lines of all the lncRNA genes (red) and all the coding-genes (green). The dashed lines indicated the thresholds selected to calculated the number of expressed lncRNA genes targeted by the Albarossa library (TPM >0.01, >0.1 or >1). **c,** Box plot showing the number of expressed lncRNA genes represented in the library across cancer cell lines with a TPM>0.1 (Top) or a TPM>1 (Bottom). Cell lines were grouped according to the tumor´s tissue of origin. n=independent cell lines per tissue type. Legend can be found below the figure. n= independent cell lines per tissue type. **d,** Sample-to-sample hierarchical clustering of the cancer cell lines using RNA-seq expression values from all the coding genes, the original lncRNA genes or, from the lncRNA genes included in the library. Rand index was calculated by comparing the cluster against the ideal cell line-tissue cluster.

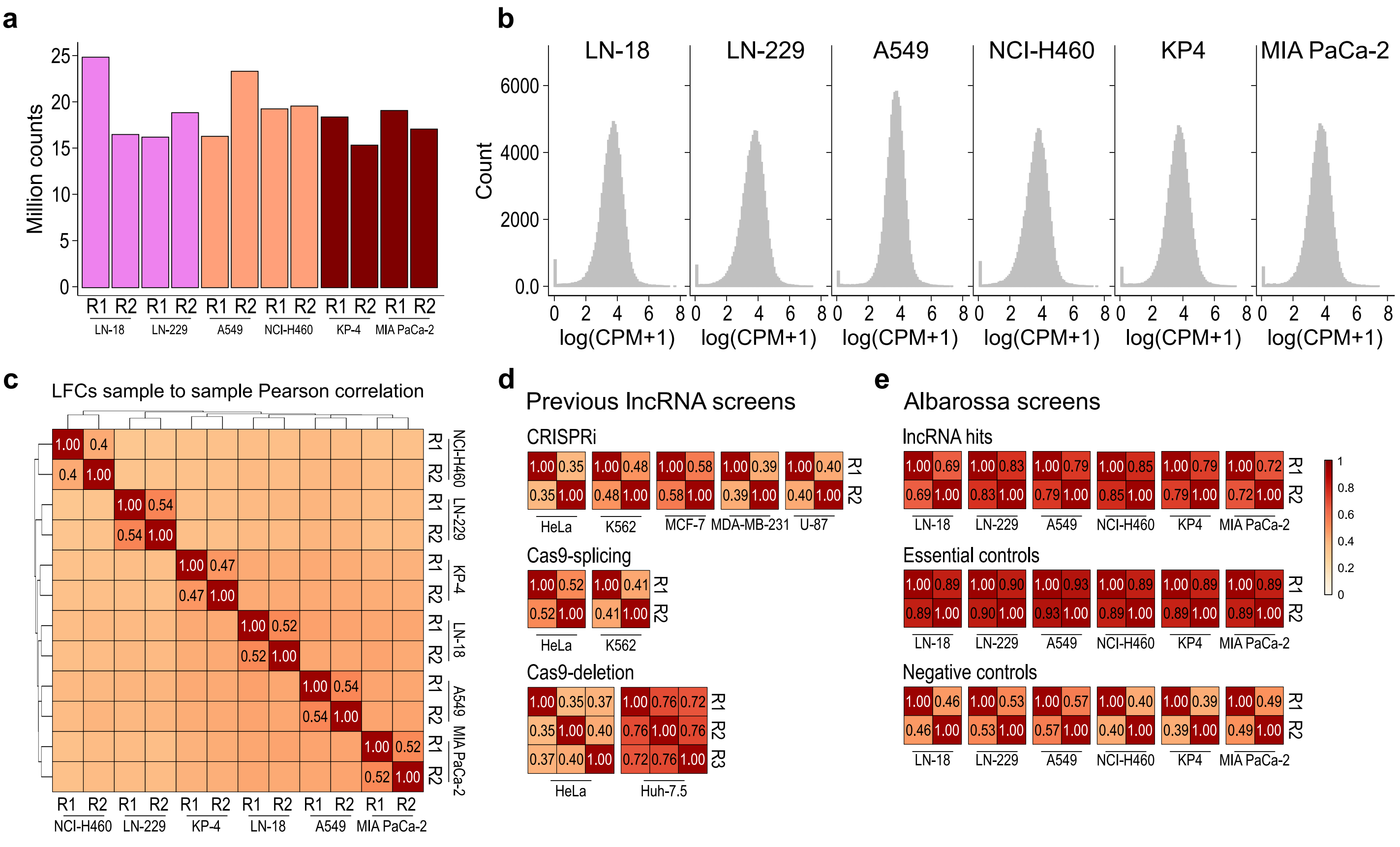

Supplementary Figure 4: Quality control of the CasRx screens.

a, After NGS, raw reads were obtained using a custom pipeline. The graphs show the number of counts in each screen replicate performed in the study. **b,** Distribution of the gRNAs average log(CPM+1) (between replicates) in each screen. CPM, counts per million. **c,** Pearson correlation between the LFC of the different samples. The values of the correlation between replicates of the same screen are indicated. **d,** Pearson correlation values between replicate LFCs of previous lncRNA LOF screens based on CRISPRi[27], Cas9-splicing site targeting[16] or Cas9-based deletion[15]. **e,** Pearson correlation values between replicate LFCs for the indicated gene subsets present in the Albarossa library in each screen. Lower replicate correlations are expected in lncRNA screes than in genome-wide perturbation of the protein-coding genome. This is due to the substantially smaller number of hits in lncRNA screens (gRNAs dropping out). The gRNAs without biological effects have more random distribution (caused by experimental noise), while perturbations with fitness effects are subjected to selective pressure, resulting in a greater correlation between replicates. Pearson correlations between screening replicates using LFC values of set of essential coding genes with are known to have biological effects (essential controls) are very high for all cell lines (0.89-0.93). Similarly, lncRNA hit replicates are highly correlated. In contrast, for negative controls (no biological relevance) display much lower correlation, as expected.

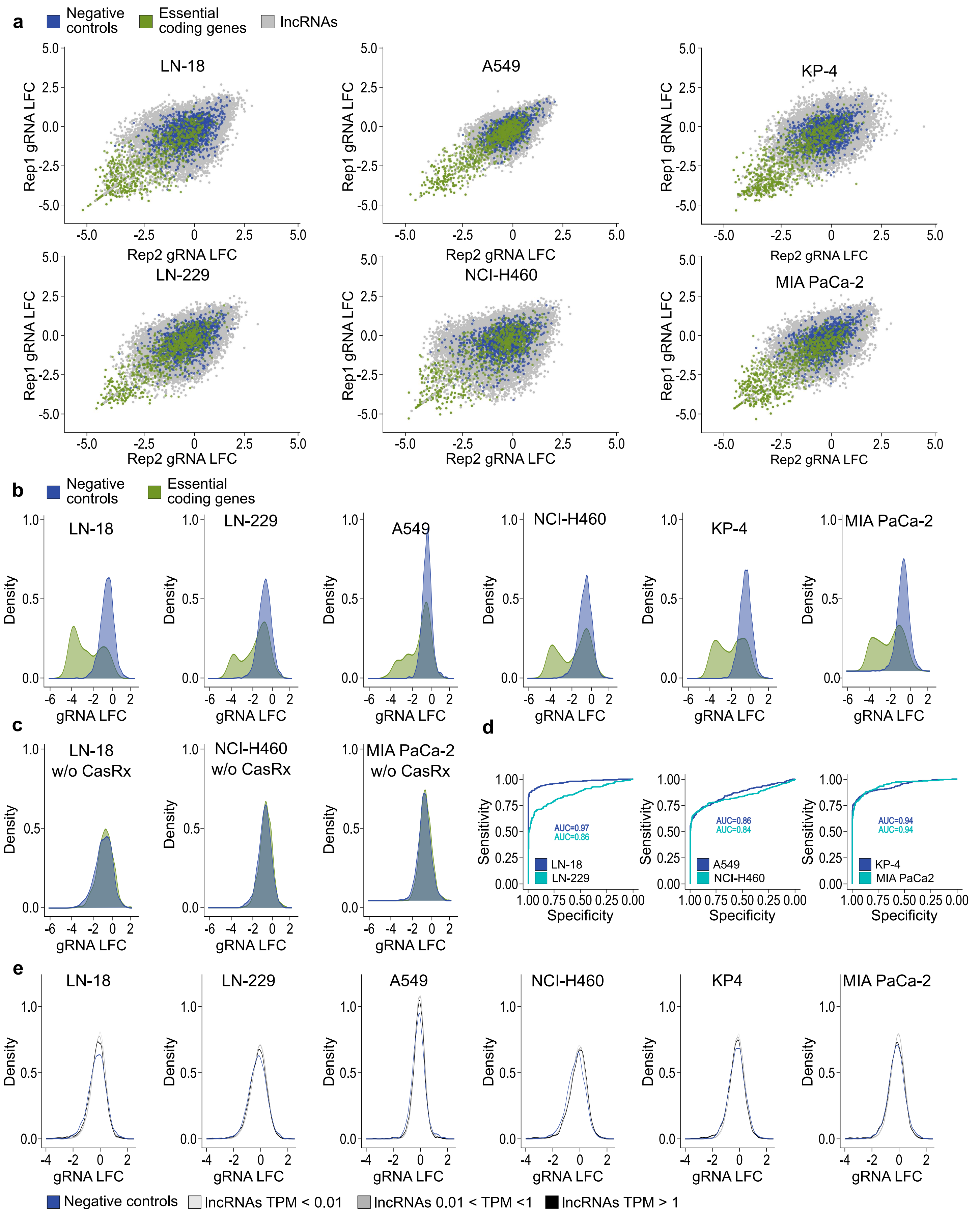
a
Negative controls
Essential coding genes
lncRNAs
LN-18
A549
KP-4
LN-229
NCI-H460
MIA PaCa-2
Rep1 gRNA LFC
Rep2 gRNA LFC
b
Negative controls
Essential coding genes
Density
gRNA LFC
c
LN-18 w/o CasRx
NCI-H460 w/o CasRx
MIA PaCa-2 w/o CasRx
d
Sensitivity
Specificity
AUC=0.97
AUC=0.86
LN-18
LN-229
AUC=0.86
AUC=0.84
A549
NCI-H460
AUC=0.94
AUC=0.94
KP-4
MIA PaCa2
e
KP4
Negative controls
lncRNAs TPM < 0.01
lncRNAs 0.01 < TPM <1
lncRNAs TPM > 1

Supplementary Figure 5: CasRx LOF fitness screens performed in six cell lines.

a, Scatter plots of gRNAs LFC between two technical replicates of all the screens. gRNAs targeting lncRNAs appear in grey, negative control gRNAs (gRNAs targeting never-essential coding genes and non-targeting gRNAs) in blue, and positive controls gRNAs (gRNAs targeting always-essential coding genes) in green. **b,** LFC distribution of negative control and positive control gRNAs of all the screens. **c,** LFC distribution of negative control and positive control gRNAs of control screens performed without CasRx expression. **d,** Receiver operating characteristic curves calculated using the average LFC of the control protein-coding genes. Always-essential genes are used as true positives and never-essential genes as false positives. The curves are shown for all the CasRx screens and the AUC values are annotated. **e,** LFC distribution of negative controls gRNAs (blue) and gRNAs targeting expressed or non-expressed lncRNA genes (scale of grey) of all the screens.

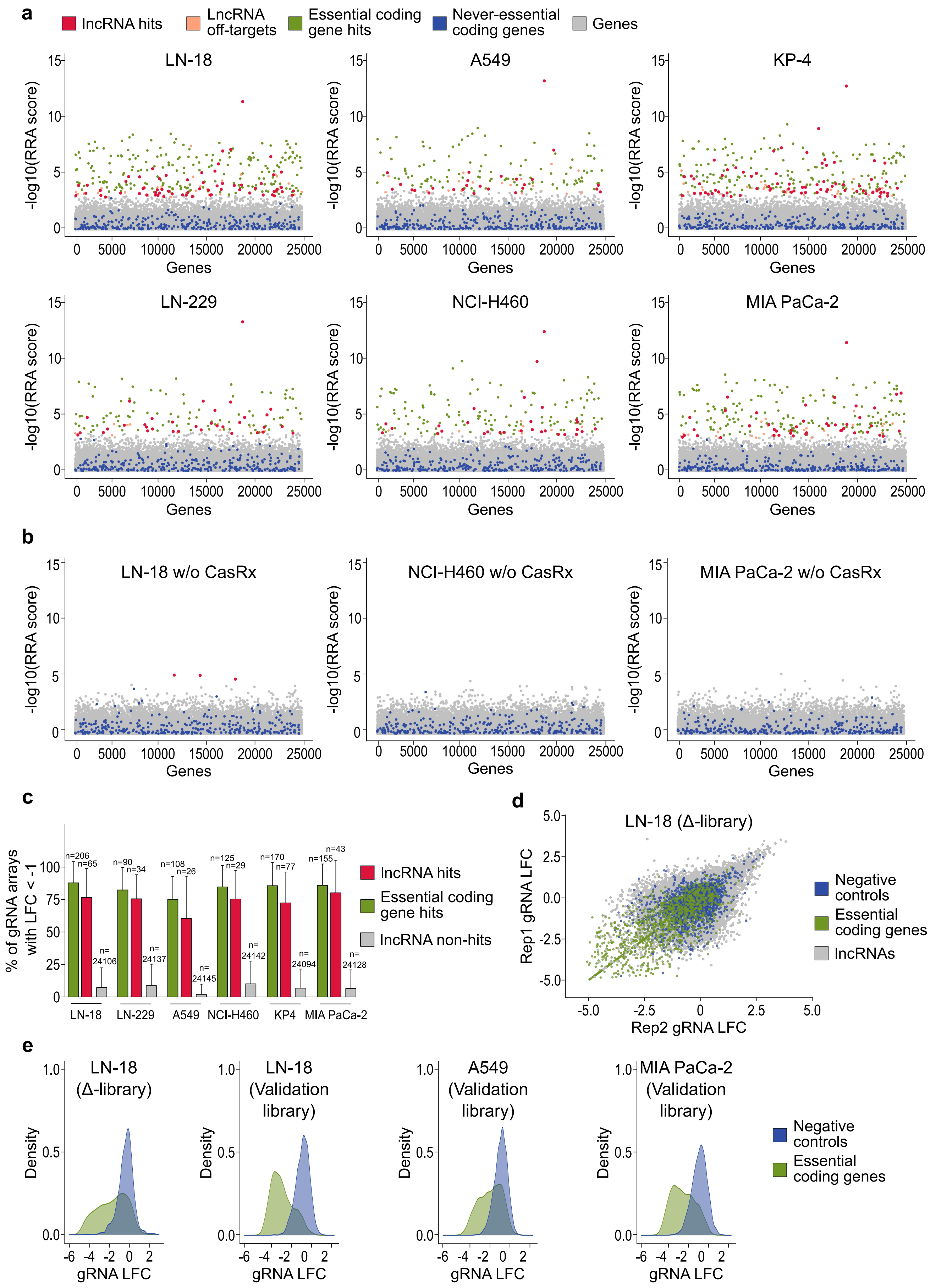

a
lncRNA hits
LncRNA off-targets
Essential coding gene hits
Never-essential coding genes
Genes
LN-18
A549
KP-4
LN-229
NCI-H460
MIA PaCa-2
-log10(RRA score)
Genes
b
LN-18 w/o CasRx
NCI-H460 w/o CasRx
MIA PaCa-2 w/o CasRx
-log10(RRA score)
Genes
c
% of gRNA arrays with LFC < -1
n=206
n=65
n=24106
n=90
n=34
n=24137
n=108
n=26
n=24145
n=125
n=29
n=24142
n=170
n=77
n=24094
n=155
n=43
n=24128
LN-18
LN-229
A549
NCI-H460
KP4
MIA PaCa-2
lncRNA hits
Essential coding gene hits
lncRNA non-hits
d
LN-18 (Δ-library)
Rep1 gRNA LFC
Rep2 gRNA LFC
Negative controls
Essential coding genes
lncRNAs
e
LN-18 (Δ-library)
LN-18 (Validation library)
A549 (Validation library)
MIA PaCa-2 (Validation library)
Density
gRNA LFC
Negative controls
Essential coding genes

Supplementary Figure 6: Filtering of off-targets and analysis of the CasRx screens.

a, Scatter plot displaying robust rank aggregation (RRA) scores for the screened genes in all performed screens calculated by MAGeCK. Never-essential coding genes are marked in blue, and significant hits are marked in red (lncRNA hits) or green (always-essential coding gene hits). The lncRNA hits that were not significantly identified after off-target gRNAs removal are marked in orange. The remainder genes are shown in grey. **b,** Scatter plot displaying robust rank aggregation (RRA) scores of screened genes, as calculated by MAGeCK of control experiments without CasRx expression. Never-essential coding genes are marked blue, and significant hits are marked red (lncRNA hits) or green (always-essential coding gene hits). Remaining genes are marked grey. **c,** Bar plot displaying the average percentage of gRNA with a LFC <-1 per individual gene. Error bars represent standard deviation. n=lncRNA genes or coding-genes. **d,** Scatter plots of gRNAs LFC between two technical screening replicates in a screen performed in the LN-18 cell line with a custom library. **e,** LFC distribution of negative control and positive control gRNAs in a screen performed in the LN-18 cell line with a custom library and in the LN-18, A549 and MIA PaCa-2 cell lines with a validation library.

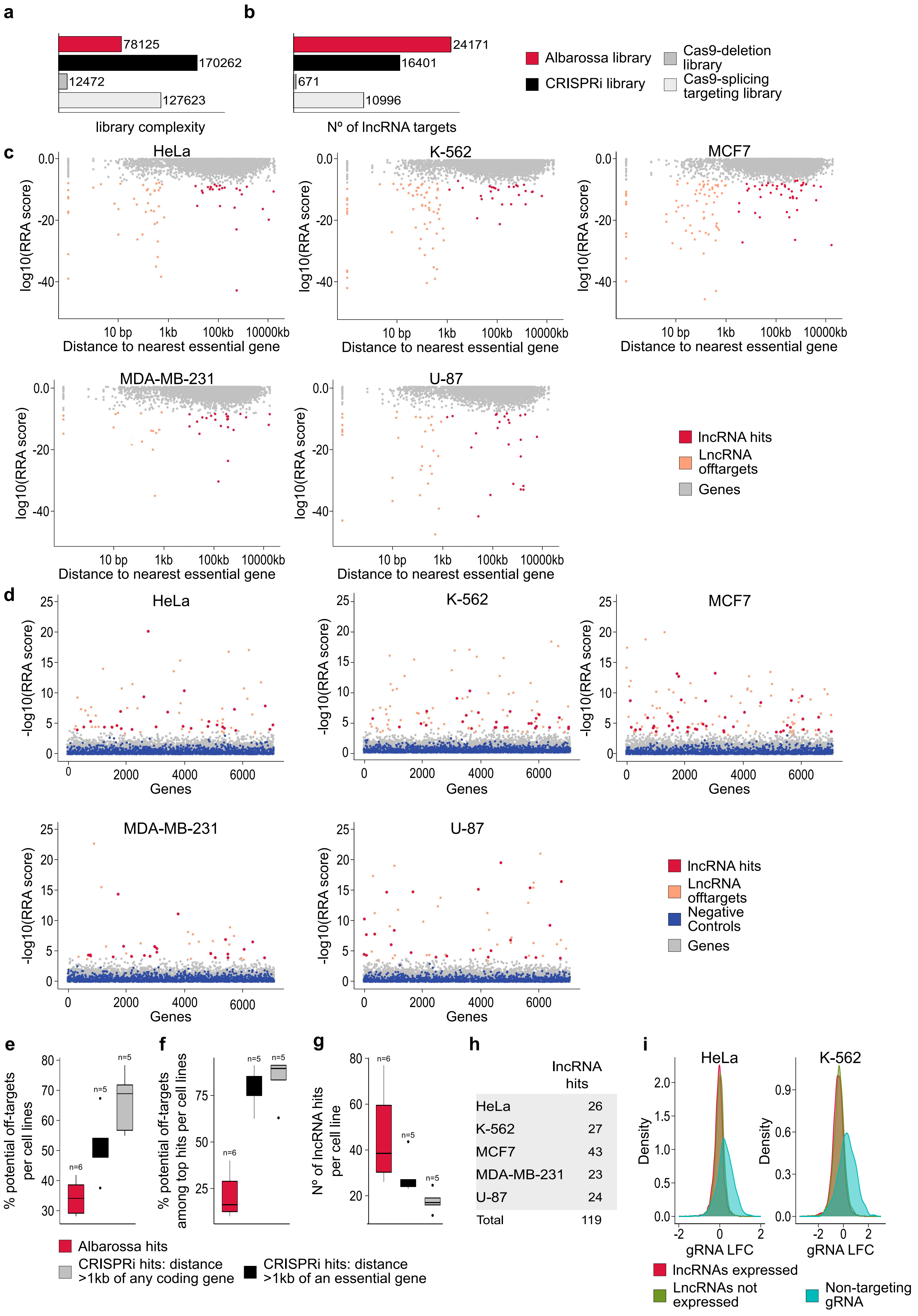

a
b
78125
170262
12472
127623
library complexity
24171
16401
671
10996
Nº of lncRNA targets
Albarossa library
CRISPRi library
Cas9-deletion library
Cas9-splicing targeting library
c
HeLa
K-562
MCF7
MDA-MB-231
U-87
log10(RRA score)
Distance to nearest essential gene
10 bp
1kb
100kb
10000kb
lncRNA hits
LncRNA offtargets
Genes
d
-log10(RRA score)
Genes
Negative Controls
e
% potential off-targets per cell lines
f
% potential off-targets among top hits per cell lines
g
Nº of lncRNA hits per cell line
n=6
n=5
Albarossa hits
CRISPRi hits: distance >1kb of any coding gene
CRISPRi hits: distance >1kb of an essential gene
h
lncRNA hits
HeLa 26
K-562 27
MCF7 43
MDA-MB-231 23
U-87 24
Total 119
i
Density
gRNA LFC
lncRNAs expressed
LncRNAs not expressed
Non-targeting gRNA

Supplementary Figure 7: Analysis of previous system screens to identify lncRNA vulnerabilities.

a, Bar plot showing the library complexity (the number of unique gRNAs) for the Albarossa library and the libraries used in the CRISPRi[27], the Cas9-based deletion[15], or the Cas9-splicing targeting screens[16]. **b,** Number of lncRNA targeted in our screening or in previous studies using different approaches[15,16,27]. **c,** Scatter plots of the robust rank aggregation (RRA) scores calculated by MAGeCK (Y-axis) and the distance to the closest essential coding gene (X-axis) of the lncRNA targeted in the CRISPRi screens. A hit was considered an off-target if a significantly detected lncRNA was <1Kb close to an essential-coding gene (orange). **d,** Scatter plot of the robust rank aggregation (RRA) scores of screened genes calculated by MAGeCK. CRISPRi screens performed in cancer cell lines are shown. Negative controls are marked in blue, and significant lncRNA hits are marked in red and, the off-target lncRNA hits are marked in orange. **e,** Box plot showing the percentage of off-target per cell line in the CasRx screens (red) or in the CRISPRi screens. n=independent cell lines. **f,** Percentage of off-target in the top hits (first quartile of the significant hits regarding FDR) per cell line in the CasRx screens (red) or in the CRISPRi screens. n=independent cell lines. **g,** Number of lncRNA hits per cell line (after off-target filtration) in line in the CasRx screens (red) or in the CRISPRi screens. n=independent cell lines. **e-g**, For the CRISPRi screens a hit was considered off-target when it was <1Kb close to an essential-coding gene (black) or to any coding gene (grey). For all the Box plot figures, the data is presented as follows: centre: median; box bounds: 25% and 75% percentile; whisker: 1.5x of inter-quartile range (IQR). Outliers are marked as independent dots. h, Summary table of all CRISPRi screens significant hits after off-target filtration. A hit was considered off-target if it was <1Kb close to an essential-coding gene. i, LFC distribution of negative control gRNAs (light blue) or gRNAs targeting expressed
