# Supplementary material for: Genome-scale pan-cancer interrogation of lncRNA dependencies using CasRx
Source: Nat Methods. 2024 Feb 26;21(4):584–96. doi: 10.1038/s41592-024-02190-0 (PMC11009108; doi:10.1038/s41592-024-02190-0)
Supplement: Supplementary file 2 — Reporting Summary [file 41592_2024_2190_MOESM2_ESM.pdf]

nature portfolio

Corresponding author(s): Roland Rad, Juan José Montero

Last updated by author(s): Nov 23, 2023

Reporting Summary

Nature Portfolio wishes to improve the reproducibility of the work that we publish. This form provides structure for consistency and transparency in reporting. For further information on Nature Portfolio policies, see our Editorial Policies and the Editorial Policy Checklist.

Statistics

For all statistical analyses, confirm that the following items are present in the figure legend, table legend, main text, or Methods section.

n/a	Confirmed	
☐	☒	The exact sample size (*n*) for each experimental group/condition, given as a discrete number and unit of measurement
☐	☒	A statement on whether measurements were taken from distinct samples or whether the same sample was measured repeatedly
☐	☒	The statistical test(s) used AND whether they are one- or two-sided<br>*Only common tests should be described solely by name; describe more complex techniques in the Methods section.*
☐	☒	A description of all covariates tested
☐	☒	A description of any assumptions or corrections, such as tests of normality and adjustment for multiple comparisons
☐	☒	A full description of the statistical parameters including central tendency (e.g. means) or other basic estimates (e.g. regression coefficient) AND variation (e.g. standard deviation) or associated estimates of uncertainty (e.g. confidence intervals)
☐	☒	For null hypothesis testing, the test statistic (e.g. *F*, *t*, *r*) with confidence intervals, effect sizes, degrees of freedom and *P* value noted<br>*Give P values as exact values whenever suitable.*
☒	☐	For Bayesian analysis, information on the choice of priors and Markov chain Monte Carlo settings
☐	☒	For hierarchical and complex designs, identification of the appropriate level for tests and full reporting of outcomes
☐	☒	Estimates of effect sizes (e.g. Cohen's *d*, Pearson's *r*), indicating how they were calculated

Our web collection on statistics for biologists contains articles on many of the points above.

Software and code

Policy information about availability of computer code

Data collection	No software was used
Data analysis	featureCounts (subread package v1.6.3), Salmon v1.5.2, limma v3.42.2, MAGeCK v0.5.9, https://gitlab.com/sanjanalab/cas13

For manuscripts utilizing custom algorithms or software that are central to the research but not yet described in published literature, software must be made available to editors and reviewers. We strongly encourage code deposition in a community repository (e.g. GitHub). See the Nature Portfolio guidelines for submitting code & software for further information.

Data

Policy information about availability of data

All manuscripts must include a data availability statement. This statement should provide the following information, where applicable:

- Accession codes, unique identifiers, or web links for publicly available datasets
- A description of any restrictions on data availability
- For clinical datasets or third party data, please ensure that the statement adheres to our policy

Data Availability
All sequencing datasets have been deposited in ENA. ENA accession number: PRJEB60776 (https://www.ebi.ac.uk/ena/browser/view/PRJEB60776).
The GENCODE Human Release 38 genome (GRCh38.p13) and transcriptome can be found here:
https://www.gencodegenes.org/human/release_38.html.

The resources from DepMap and the Cancer Cell Line Encyclopedia (CCLE) can be found here:
https://doi.org/10.6084/m9.figshare.19700056.v2.
The resources from RNAcentral can be found here:
https://ftp.ebi.ac.uk/pub/databases/RNAcentral/releases/16.0.

Human research participants

Policy information about studies involving human research participants and Sex and Gender in Research.

Reporting on sex and gender	n/a
Population characteristics	n/a
Recruitment	n/a
Ethics oversight	n/a

Note that full information on the approval of the study protocol must also be provided in the manuscript.

Field-specific reporting

Please select the one below that is the best fit for your research. If you are not sure, read the appropriate sections before making your selection.

☒ Life sciences ☐ Behavioural & social sciences ☐ Ecological, evolutionary & environmental sciences

For a reference copy of the document with all sections, see nature.com/documents/nr-reporting-summary-flat.pdf

Life sciences study design

All studies must disclose on these points even when the disclosure is negative.

Sample size	The sample size is reported in the figure legends for each individual experiment. We have no any kind of experiments (as animals base ones) that requiered sample size calculations. Most of our experiments are screenings, with intenal controls and independent gRNAs that validate the experiment statistically. For other kind of experiments (lncRNA validations), we validate each lncRNA with several different methods (colony formation, proliferation curves and RNA-seq). All the experiments behave in the same way, making the need of extra sample size innecesary.
Data exclusions	No data was excluded.
Replication	All attempt of replication were successful. How many times each experiment was replicates is reported in the figure legends of each individual experiment.
Randomization	Most of our experiments included internal controls, making randomizing the samples unnecessary. The final outputs are independent of the researcher, as NGS is inherently unbiased.
Blinding	Most of our experiments included internal controls, making blinding unnecessary. The final outputs are independent of the researcher, as NGS is inherently unbiased.

Reporting for specific materials, systems and methods

We require information from authors about some types of materials, experimental systems and methods used in many studies. Here, indicate whether each material, system or method listed is relevant to your study. If you are not sure if a list item applies to your research, read the appropriate section before selecting a response.

Materials & experimental systems

n/a	Involved in the study	
☒	☐	Antibodies
☐	☒	Eukaryotic cell lines
☒	☐	Palaeontology and archaeology
☒	☐	Animals and other organisms
☒	☐	Clinical data
☒	☐	Dual use research of concern

Methods

n/a	Involved in the study	
☒	☐	ChIP-seq
☐	☒	Flow cytometry
☒	☐	MRI-based neuroimaging

Eukaryotic cell lines

Policy information about cell lines and Sex and Gender in Research

Cell line source(s)	HEK-293T, KP-4 and MIA PaCa-2 were available in our research center. A549 and NCI-460 were provided by Mariano Barbacid from the Spanish National Cancer Research Center (CNIO). LN18 and LN-229 were provided by massimo Squatrito from the Spanish National Cancer Research Center (CNIO).
Authentication	None of the cell lines were authenticated.
Mycoplasma contamination	All the cell lines were tested regularly for mycoplasma contamination. And all of them were negative.
Commonly misidentified lines (See ICLAC register)	None of the cell lines were authenticated.

Flow Cytometry

Plots

Confirm that:

☒ The axis labels state the marker and fluorochrome used (e.g. CD4-FITC).

☒ The axis scales are clearly visible. Include numbers along axes only for bottom left plot of group (a 'group' is an analysis of identical markers).

☒ All plots are contour plots with outliers or pseudocolor plots.

☒ A numerical value for number of cells or percentage (with statistics) is provided.

Methodology

Sample preparation	Cells were resuspended in FACS Buffer (PBS, 3% FBS, 5mM EDTA).
Instrument	Beckman Coulter Cytoflex LX system.
Software	FlowJo v10.7.2
Cell population abundance	A minimum of 10000 cells were recorded.
Gating strategy	Singles were selected using FSC-width and FSC-A values, tRFP657 cells were selectd using SSC-A and Y675-PC5-A values, finally GFP intensity in the tRFP657 positives cells were measured as the mean intensity of the B525-FITC-A values.

☒ Tick this box to confirm that a figure exemplifying the gating strategy is provided in the Supplementary Information.
