# Supplementary material for: Genome-scale pan-cancer interrogation of lncRNA dependencies using CasRx
Source: Nat Methods. 2024 Feb 26;21(4):584–96. doi: 10.1038/s41592-024-02190-0 (PMC11009108; doi:10.1038/s41592-024-02190-0)
Supplement: Supplementary file 5 — Supporting data for Supplementary Fig. 1b. [file 41592_2024_2190_MOESM5_ESM.pdf]

HyPBase PCR

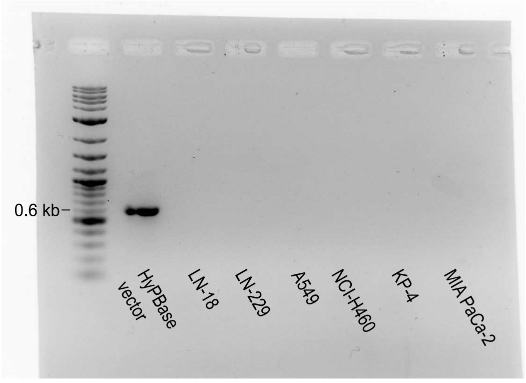

CasRx PCR

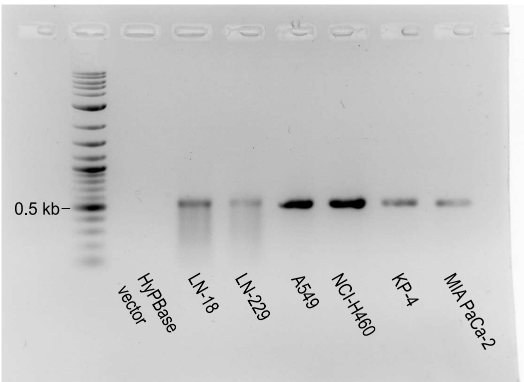

Source data Fig. 1: Full blot from the Supplementary Figure 1b.

Agarose gel electrophoresis displaying the PCR amplification of the HypBase-transposase or the CasRx-transposon locus using either the HypBase-transposase vector or DNA extracted from the CasRx clones as template.
